# Dataset on the existence of andisols under aridic-hyperthermic environments in the harrats region of the Arabian Shield

**DOI:** 10.1016/j.dib.2019.105072

**Published:** 2020-01-03

**Authors:** Magboul M. Sulieman, Abdelazeem Sh Sallam, Abdullah S. Al-farraj, Eric C. Brevik

**Affiliations:** a Soil Sciences Department, College of Food and Agricultural Sciences, King Saud University, P.O. Box 2460, Riyadh, 11451, Saudi Arabia; b Department of Soil and Environment Sciences, Faculty of Agriculture, University of Khartoum, Khartoum North, 13314, Shambat, Sudan; c Department of Natural Sciences, Dickinson State University, Dickinson, ND, USA; d Department of Agriculture and Technical Studies, Dickinson State University, Dickinson, ND, USA

**Keywords:** Volcanic soils, Aridic-hyperthermic environments, Poorly-crystalline minerals, Arabian shield, Andic soil properties

## Abstract

The data from twelve representative soil profiles on six harrats (two profiles from each Harrat) within the Arabian Shield are presented, including full morphological descriptions made during the field the soil survey. A number of selected physicochemical and mineralogical analyses were also conducted in the laboratory and the data were interpreted to examine the possibility of the presence of andic/vitric soil properties in the studied soils, and thus the existence of Andisols in the harrats soils. The existence of andic/vitric properties in soils is not typical of regions characterized by aridic and hyperthermic soil moisture and temperature regimes, respectively, and is probably due to the influence of paleoclimatic conditions. The data is available online for further reuse and to provide a better understanding of the findings linked to this research.

**Table:** Specifications Table

Subject	Soil science: Pedology.
More specific subject area	Soil classification.
Type of data	Table, image.
How data were acquired	The field data for the morphological properties of the representative profiles were acquired during a soil survey, carried out in April-2017 using standard guidelines for soil profile description. Data on the physicochemical properties were acquired using standard soil laboratory methods, while the clay mineralogical composition data were acquired using selective dissolutions, XRD, TG, ATR/FTIR, SEM and TEM techniques.
Data format	Raw, analysed
Experimental factors	Soil samples were air dried (20–22 °C), ground (excluding rock fragments and concretions), screened through a 2 mm sieve and divided into representative subsamples using a riffle splitter.
Experimental features	The physicochemical soil samples from the different horizons (2 samples from each horizon resulting in a total of 102 samples)of the representative profiles were analysed using standard soil laboratory methods, while selected soil samples were analysed for mineralogical composition using selective dissolutions, XRD, TG, ATR/FTIR, SEM and TEM techniques and linked to the paleoclimatic conditions in the area to examine the presence of andic/vitric soil properties and thus the existence of Andisols.
Data source location	Harrat Ithnayn (26°46ʹ76ʺ N, 40°03ʹ55ʺ E), Harrat Kurma (24°31′60″ N, 40°7′60″ E), Harrat Rahat (24°25ʹ28ʺ N, 39°31ʹ06ʺ E), Harrat Khaybar (25°44ʹ04ʺ N, 39°58ʹ51ʺ E), Harrat Kishb (23°12ʹ00ʺ N, 41°20ʹ14ʺ E) and Harrat Hutaymah (26°58′48″ N, 42°24′00″ E), Arabian Shield, Saudi Arabia.
Data accessibility	The data is provided in this article.
Related research article	Magboul Sulieman, Abdelazeem Sallam, Abdullah Al-farraj, Eric Brevik. First evidence for the presence of Andisols in the dry-hot environment of the Arabian Shield. Geoderma 2019. (https://doi.org/10.1016/j.geoderma.2019.114068).

**Table:** 

**Value of the Data** • The data showed that andic/vitric soil properties and poorly-crystalline minerals were present in soils of the harrats region, and the soils were thus classified as Andisols. • The data is a valuable resource for pedologists and soil scientists in general to gain a better understanding of the role of paleoclimate on soil formation. • The dataset can be considered a guideline and a point of comparison for future research on soils in similar geoenvironmental settings around the world. • The presence of Andisols in the studied area was attributed to the influence of the paleoclimatic conditions. • The data given in this article is a brief explanation of the data attributed to the research article titled “First evidence for the presence of Andisols in the dry-hot environment of the Arabian Shield” which accepted in 2019 by Geoderma (https://doi.org/10.1016/j.geoderma.2019.114068).

## 1 Data

**Fig. 1:**
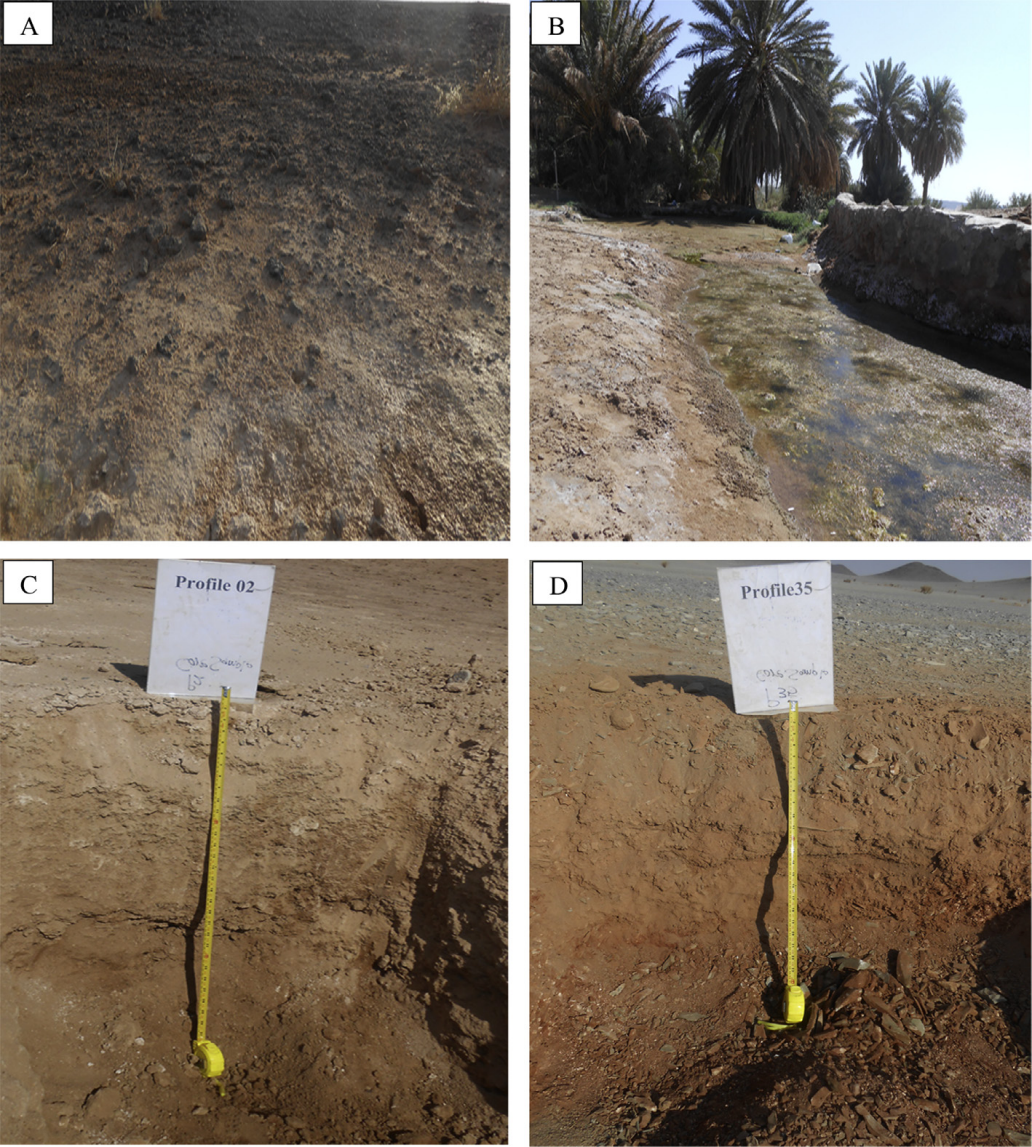
An overview of some of the soil features in the study area showing; a surface cover of basalt fragments (A), irrigated date palm farm with saline water from the main weel (B), profile HPN02 on the sabkha landform (C) and profile HPN35 in a hillslope position (D).

Fig. 1 gives an overview of some of the features and profiles in the study area. Table 1 gives detailed information for selected environmental characteristics of the selected representative soil profiles in the study area. Table 2 presents the morphological descriptions of the representative soil profiles. Table 3, Table 4 illustrate the physicochemical properties data for the representative soil profiles. Table 5 shows data from the selective dissolutions analysis and P-retention of the representative soil profiles. Table 6 shows data on the most important ratios of selective dissolutions analysis and calculated allophane and ferrihydrite concentrations for the studied profiles. Table 7 presents the index values for the andic soil properties in the studied representative profiles.

**Table 1:** Site characteristics for selected representative profiles from different harrats within the Arabian Shield, Saudi Arabia.

Site	Profile	Coordinates WGS 84 (N/E)	Elevation m a.s.l.	Parent material	Land form /position	Geological age	Natural Vegetation	Land use	Land cover
Harrat Kishb	HKP01	22.2875/41.0672	1015	Basaltic rocks	Ap	2.4 ± 0.8 Ma	Acacia spp.	Range land	Rock fragments
	HKP02	22.6809/41.1510	1013	Basaltic rocks	Lv	2.4 ± 0.8 Ma	Acacia spp.	Range land	Rock fragments
Harrat Rahat	HRP03	23.1316/39.5911	1031	Basaltic rocks	Lv	10 Ma	Acacia spp.	Range land	Rock fragments
	HRP04	24.2843/39.4543	775	Basaltic rocks	Lv	10 Ma	Acacia spp.	Range land	Rock fragments
Harrat Kurma	HKuP05	24.3668/40.1980	759	Basaltic rocks	Ap	20 ± 2 Ma	Acacia spp.	Range land	Rock fragments
	HKuP06	24.2809/40.4960	817	Basaltic rocks	As,Dg	20 ± 2 Ma	Acacia spp.	Range land	Rock fragments
Harrat Khaybar	HKhP07	25.3810/42.2324	1112	Basaltic rocks	As,Dg	5 Ma	Acacia spp.	Range land	Rock fragments
	HKhP08	25.3811/39.1926	764	Basaltic rocks	Lv	5 Ma	Acacia spp.	Range land	Rock fragments
Harrat Ithanyn	HThP09	26.1662/40.2090	1172	Basaltic rocks	Lv	3 Ma	Acacia spp.	Range land	Rock fragments
	HThP10	26.1739/40.1660	1169	Basaltic rocks	Lv	3 Ma	Acacia spp.	Range land	Rock fragments
Harrat Hutaymah	HHuP11	26.9415/42.0980	1054	Basaltic rocks	Pr	1.8 Ma	Acacia spp.	Range land	Rock fragments
	HHuP12	26.3380/41.4020	1050	Basaltic rocks	Pp,Pt	1.8 Ma	Acacia spp.	Range land	Rock fragments

Symbols: Ap = alluvial plain, Lv = lava field, Sbkh = Sabkha, As, Dg = active slope, Pp, Pt = pediplain with deep soils, Pr = pediplain with shallow soils.

**Table 2:** Morphological properties of selected representative profiles from different harrats of the Arabian Shield.

Profile	Horizon	Depth (cm)	Matrix color Munsell moist	Texture^a^	Structure^b^	Roots^c^	HCl effervescence^d^	Consistence^e^ Moist	Biological activity^f^	Horizon Boundary^g^
				Field						
HKP01	A	0–10	7.5 YR 3/3	S	SG	1	1	VF	M	C, SM
	Bw	10–25	7.5 YR 3/4	SL	1, M, ABK	1	1	FR	W	C, SM
	Bw1	25–55	7.5 YR 3/4	SL	1, M, ABK	1	1	FR	W	C, SM
	Bw2	55–80	7.5 YR 3/4	SL	1, M, ABK	1	2	FR	W	
HKP02	A	0–15	10 YR 3/2	SL	1, M, GR	1	3	FR	M	C, SM
	Bw1	15–45	7.5 YR 3/4	SL	1, M, ABK	1	3	FR	W	C, SM
	Bw2	45–70	7.5 YR 3/4	SL	1, M, ABK	1	2	FR	W	C, SM
	C	70+	7.5 YR 3/4	SL	MA	0	3	FR	W	
HRP03	A	0–10	10 YR 3/2	SL	MA	1	1	VF	W	C, SM
	AB	10–40	2.5 YR 2.5/2	SL	MA	0	2	VF	N	A, SM
	Bw	40–65	10 YR 3/1	LS	MA	0	2	VF	N	A, SM
	Bw2	65–115	2.5 YR 3/2	SL	MA	0	1	VF	N	A, SM
	C	115–130	7.5 YR 2.5/2	LS	MA	0	1	VF	N	
HRP04	A	0–20	7.5 YR 4/4	SCL	1, F, SBK	1	1	FR	W	C, SM
	Bw	20–66	7.5 YR 4/4	L	2, M, GR	1	3	FR	N	D, SM
	C	66–95	7.5 YR 4/4	L	1, M, GR	0	1	FR	N	
HKuP05	A	0–23	7.5 YR 3/4	L	2, M, GR	2	1	VF	M	C, W
	Bw1	23–45	10 YR 4/4	SL	1, M, SBK	1	2	FR	W	C, W
	Bw2	45–60	10YR 3/2	LS	2, M, ABK	0	1	VF	N	C, W
	C	60+	10YR 3/4	SL	1, M, SBK	0	2	FR	N	
HKuP06	A	0–5	7.5 YR 3/4	LS	2, F, GR	2	1	FR	M	C, SM
	Bw1	5–20	10 YR 4/4	SL	1, M, SBK	1	2	FR	M	C, SM
	Bw2	20–50	10YR 3/2	SL	1, M, ABK	1	3	FR	W	C, SM
	C1	50–62	10YR 3/2	SL	1, M, ABK	0	4	FR	N	C, SM
	C2	62+	7.5 YR 4/4	L	2, M, ABK	0	4	VF	N	
HKhP07	A	0–22	5YR 3/4	C	1, F, SBK	1	1	VF	M	A, SM
	Bw	22–47	5YR 3/3	CL	3, M, SBK	1	3	VF	W	C, SM
	C	47–75	2.5YR 3/4	SCL	1, F, SBK	1	1	FR	W	D, IR
	C2	75–110	5YR 3/4	L	1, F, SBK	1	1	VF	W	
HKhP08	C	0–25	7.5 YR 4/6	C	2, M, SBK	1	1	VF	M	D, SM
	ABz	25–45	10 YR 4/6	C	MA	0	3	VF	W	A, SM
	Cz	45–70	5 YR 3/4	C	1, F, AB	1	1	VF	W	D, SM
	2Bwz	70–95	2.5 YR 4/2	CL	MA	0	2	VF	W	D, SM
	2Cz	95–120	10 YR 3/3	CL	MA	0	2	VF	W	C, SM
	2C2	120+	2.5 YR 5/4	CL	MA	1	1	VF	W	
HThP09	A	0–15	10YR 3/2	SL	1, M, GR	3	3	FR	M	C, SM
	Bw1	15–35	10YR 3/2	SL	1, F, ABK	2	3	VR	W	C, SM
	Bw2	35–60	10YR 3/2	SL	1, M, ABK	1	3	FR	W	C, SM
	C1	60–85	10YR 3/2	SL	MA	0	2	FR	W	C, SM
	C2	85+	7.5 YR 3/4	L	MA	0	1	VF	N	
HThP10	A	0–15	10YR 3/2	SL	1, M, GR	1	2	FR	M	C, SM
	Bw1	15–35	10YR 3/2	SL	1, F, ABK	1	2	FR	M	C, SM
	Bw2	35–50	10YR 3/2	SL	1, M, ABK	0	2	FR	W	
HHuP11	A	0–10	10 YR 4/4	LS	1, F, ABK	1	1	FR	W	C, SM
	Bw1	10–38	10 YR 3/6	LS	2, F, ABK	0	1	FR	N	D, SM
	Bw2	38–70	10 YR 3/6	CL	2, F, ABK	0	2	VF	N	C, SM
	C	70+	7.5 YR 4/4	SC	3, M, SBK	0	1	FR	N	
HHuP12	A	0–20	5 YR 3/4	LS	1, F, ABK	1	2	FR	W	D, SM
	Bw1	20–45	5 YR 3/4	LS	2, F, ABK	0	2	FR	N	D, SM
	Bw2	45–75	7.5 YR 3/4	SL	2, F, ABK	0	1	FR	N	D, SM
	C	75+	5 YR 3/4	LS	3, M, SBK	0	1	FR	N	

a Field texture: S = Sand; SL = sandy loam; LS = loamy sand; SCL = sandy clay loam; L = loam; CL = clay loam; C = clay.

b Structure: 1 = weak; 2 = moderate; 3 = strong; F = fine; M = medium; C = coarse; SBK = subangular blocky; ABK = angular blocky; GR = granular; SG = Single grain; MA = massive.

c Roots abundance: 0 = none; 1 = few (2–20%); 2 = common (20–50%); 3 = many (>50%).

d HCl effervescence: 1 = slight; 2 = moderate; 3 = strong; 4 = very strong.

e Consistence: FR = firm; VF = very firm .

f Biological activity: N = none; W = weak; M = moderate.

g Boundary: C = clear; D = diffuse; A = abrupt; SM = smooth; IR = irregular.

**Table 3:** Texture, particle size distribution, water retention, bulk density and soil pH by horizon in the selected representative profiles from different harrats of the Arabian Shield.

Profile	Horizon	Depth cm	Sand	Silt	Clay			Textural Class	Water retention		BD^b^	pH			
			50–2000 μm	2–50 μm	<2 μm	1–2 μm	0.1–1 μm								
			g kg⁻^1^					NRCS, USDA^a^	33 kPa (%)	15,00 kPa (%)	Mg m⁻^3^	H_2_O	CaCl_2_	KCl	NaF
HKP01	A	0–10	832.4	120.7	46.90	12.80	34.10	Loamy Fine Sand	4.42	0.87	1.60	8.85	8.12	7.98	9.72
	Bw	10–25	729.3	186.9	83.80	22.80	61.00	Sandy Loam	4.62	0.99	1.60	9.07	8.20	8.06	9.43
	Bw1	25–55	549.7	335.3	115.0	31.30	83.70	Sandy Loam	11.1	1.14	1.61	9.07	8.11	7.75	9.80
	Bw2	55–80	663.6	228.0	108.4	29.50	78.90	Sandy Loam	12.6	1.17	1.61	8.96	8.09	7.65	10.0
HKP02	A	0–15	740.8	182.2	77.00	20.90	56.10	Sandy Loam	11.6	1.12	1.59	9.10	8.15	7.85	9.88
	Bw1	15–45	468.0	473.8	58.20	15.80	42.40	Sandy Loam	6.68	1.22	1.61	8.35	8.32	8.16	9.95
	Bw2	45–70	666.8	296.6	36.60	10.00	26.60	Sandy Loam	25.9	1.31	1.62	8.13	8.07	7.66	9.87
	C	70+	689.6	275.5	34.90	9.500	25.40	Sandy Loam	17.2	1.13	1.62	7.93	7.98	7.66	9.92
HRP03	A	0–10	800.0	50.00	150.0	40.50	109.5	Sandy Loam	14.8	7.67	1.49	8.77	7.88	7.72	9.46
	AB	10–40	787.5	61.20	151.3	40.85	110.5	Sandy Loam	8.43	4.13	1.45	8.91	7.96	7.77	9.14
	Bw	40–65	859.1	28.20	112.7	30.43	82.27	Loamy Sand	6.99	3.72	1.53	8.60	8.00	7.64	9.67
	Bw2	65–115	775.0	37.50	187.5	50.63	136.9	Sandy Loam	6.32	3.85	1.54	8.56	8.05	7.59	9.97
	C	115–130	887.5	12.50	100.0	27.00	73.00	Loamy Sand	10.2	4.69	1.54	8.65	8.15	7.89	9.85
HRP04	A	0–20	500.0	242.5	257.5	69.53	188.0	Sandy Clay Loam	10.5	4.79	1.53	8.31	8.11	7.95	9.69
	Bw	20–66	337.5	437.5	225.0	60.75	164.3	Loam	6.97	4.03	1.54	8.36	8.12	7.93	9.30
	C	66–95	290.0	447.5	262.5	70.88	191.6	Loam	12.1	5.68	1.54	8.53	8.15	7.77	9.82
HKuP05	A	0–23	433.1	301.8	265.1	87.10	178.0	Loam	16.2	7.41	1.58	8.46	8.14	7.75	9.91
	Bw1	23–45	700.7	228.3	71.00	17.90	53.00	Sandy Loam	10.2	3.91	1.62	7.84	7.97	7.71	10.0
	Bw2	45–60	756.8	195.0	48.20	12.60	35.60	Loamy Fine Sand	7.84	3.04	1.62	7.99	8.00	7.82	9.80
	C	60+	737.7	212.6	49.70	12.30	37.40	Sandy Loam	8.55	3.22	1.62	8.02	8.00	7.80	9.95
HKuP06	A	0–5	809.1	134.1	56.80	16.90	39.80	Loamy Fine Sand	6.22	2.97	1.58	8.30	7.7	7.83	9.47
	Bw1	5–20	663.1	256.3	80.50	22.10	58.50	Sandy Loam	11.2	4.32	1.61	8.79	8.04	7.72	10.0
	Bw2	20–50	632.8	304.8	62.40	15.70	46.70	Sandy Loam	11.5	4.21	1.61	8.74	8.01	7.62	10.1
	C1	50–62	543.1	369.9	87.00	22.60	64.40	Sandy Loam	13.3	4.65	1.61	8.80	8.07	7.62	10.3
	C2	62+	352.8	486.3	160.9	36.40	124.4	Loam	17.6	5.43	1.61	8.77	8.14	7.62	10.3
HKhP07	A	0–22	350.0	171.2	478.8	129.3	349.5	Clay	14.1	5.68	1.51	8.11	8.00	7.84	9.58
	Bw	22–47	450.0	218.8	331.2	89.42	241.8	Clay Loam	14.9	4.80	1.51	8.23	8.05	7.86	9.23
	C	47–75	460.0	215.0	325.0	87.75	237.3	Sandy Clay Loam	17.0	4.48	1.44	8.25	8.10	7.72	9.77
	C2	75–110	455.0	270.0	275.0	74.25	200.8	Loam	13.7	4.70	1.49	8.19	8.15	7.69	10.1
HKhP08	C	0–25	350.0	150.0	500.0	135.0	365.0	Clay	20.5	12.2	1.54	8.23	7.93	7.77	9.51
	ABz	25–45	300.0	300.0	400.0	108.0	292.0	Clay	17.1	8.92	1.53	8.90	7.95	7.76	9.13
	Cz	45–70	150.0	350.0	500.0	135.0	365.00	Clay	17.0	8.82	1.53	8.19	8.02	7.64	9.69
	2Bwz	70–95	350.0	375.0	275.0	74.25	200.75	Clay Loam	16.2	7.69	1.52	8.24	8.00	7.54	9.92
	2Cz	95–120	352.6	300.0	347.4	93.80	253.60	Clay Loam	21.0	12.8	1.52	8.40	8.05	7.79	9.75
	2C2	120+	615.0	110.0	275.0	74.25	200.75	Clay Loam	19.4	9.81	1.54	8.23	8.02	7.74	9.57
HThP09	A	0–15	525.8	354.0	120.3	35.10	85.20	Sandy Loam	13.7	5.10	1.59	8.72	7.99	7.50	10.1
	Bw1	15–35	590.8	315.7	93.50	27.20	66.30	Sandy Loam	12.5	4.75	1.61	8.81	8.08	7.51	10.1
	Bw2	35–60	535.5	384.3	80.30	23.20	57.10	Sandy Loam	13.4	4.55	1.61	8.92	8.14	7.46	10.1
	C1	60–85	535.1	354.2	110.8	35.10	75.60	Sandy Loam	13.5	4.98	1.62	8.99	8.15	7.42	9.97
	C2	85+	500.5	378.4	121.1	35.60	85.50	Loam	14.2	5.06	1.62	9.09	8.14	7.42	10.0
HThP10	A	0–15	523.8	402.2	74.00	17.10	56.90	Sandy Loam	13.6	4.44	1.55	7.85	8.02	7.79	10.3
	Bw1	15–35	542.7	362.8	94.60	24.40	70.20	Sandy Loam	13.3	4.75	1.56	8.00	8.00	7.76	10.3
	Bw2	35–50	587.0	328.9	84.10	21.40	62.70	Sandy Loam	12.5	4.62	1.58	7.94	7.95	7.72	10.2
HHuP11	A	0–10	680.0	285.0	35.00	9.500	25.50	Sandy Loam	17.2	1.13	1.57	7.93	8.09	7.56	9.87
	Bw1	10–38	586.0	364.5	49.50	13.50	36.00	Sandy Loam	18.8	1.50	1.58	8.22	8.22	7.59	9.98
	Bw2	38–70	215.5	441.7	342.8	93.20	249.6	Clay Loam	5.83	4.07	1.60	7.38	8.08	7.23	10.1
	C	70+	340.0	2870	373.0	101.5	271.5	Clay Loam	15.6	7.50	1.61	7.43	8.28	7.40	10.1
HHuP12	A	0–20	330.0	350.0	320.0	87.00	233.0	Clay Loam	17.6	6.43	1.53	7.67	8.14	7.55	10.1
	Bw1	20–45	320.0	362.0	318.0	86.50	231.5	Clay Loam	18.4	7.02	1.54	7.68	8.13	7.62	9.87
	Bw2	45–75	350.0	350.0	300.0	81.60	218.4	Clay Loam	19.5	8.30	1.59	7.89	8.20	7.90	10.2
	C	75+	842.0	80.00	78.00	21.20	56.80	Loamy Fine Sand	18.6	9.33	1.60	8.95	8.41	8.21	10.2

a Natural Resources Conservation Services at United State Department of Agriculture.

b Bulk density using core method.

**Table 4:** Additional chemical properties of the selected representative profiles from different harrats of the Arabian Shield.

Profile	Horizon	Depth cm	ΔpH			Exchangeable cations (cmol_c_ kg⁻^1^)				CEC-7^a^	BS^b^	BS^c^	EC^d^	CCE^e^	TOM^f^	Gypsum
			H_2_O–CaCl_2_	H_2_O–KCl	H_2_O–NaF	Na^+^	K^+^	Ca^2+^	Mg^2+^	cmol_c_ kg^⁻1^	%		dS m^−1^	g kg^−1^		
HKP01	A	0–10	0.73	0.87	−0.87	3.34	1.51	40.0	3.00	8.25	98.97	521.2	0.22	19.4	7.20	4.40
	Bw	10–25	0.87	1.01	−0.36	4.24	1.38	41.0	1.00	10.16	95.18	413.4	0.16	25.3	6.18	1.10
	Bw1	25–55	0.96	1.32	−0.73	0.22	0.63	32.0	23.0	19.57	99.11	281.0	0.15	25.4	5.48	19.2
	Bw2	55–80	0.87	1.31	−1.07	0.23	0.65	24.0	34.0	32.07	99.16	180.9	0.17	40.7	5.45	1.60
HKP02	A	0–15	0.95	1.25	−0.78	0.18	0.46	45.0	7.00	16.30	97.59	319.0	0.16	44.2	8.87	1.10
	Bw1	15–45	0.03	0.19	−1.60	71.9	1.38	226.0	24.0	23.37	99.75	1069.8	22.8	81.7	5.75	1.10
	Bw2	45–70	0.06	0.47	−1.74	21.5	0.91	64.0	63.0	28.80	99.53	441.0	9.68	71.7	3.38	40.5
	C	70+	−0.05	0.27	−1.99	38.9	1.27	159.0	23.0	27.72	99.64	656.6	16.3	46.2	5.45	30.7
HRP03	A	0–10	0.89	1.05	−0.69	0.25	1.90	20.5	110.0	8.83	98.45	1477.9	0.77	0.58	2.07	2.80
	AB	10–40	0.95	1.14	−0.23	0.30	2.05	27.3	27.0	14.15	97.78	383.8	1.10	2.06	4.14	2.50
	Bw	40–65	0.60	0.96	−1.07	0.80	1.15	27.5	105.0	15.17	98.58	873.4	2.75	7.94	0.34	6.30
	Bw2	65–115	0.51	0.97	−1.41	1.12	0.45	29.5	65.0	16.78	97.45	563.2	2.65	2.20	0.17	4.20
	C	115–130	0.50	0.76	−1.20	1.11	0.43	22.0	58.0	7.23	96.65	1106.5	2.79	0.15	0.34	3.50
HRP04	A	0–20	0.20	0.36	−1.38	3.34	1.51	40.0	3.00	10.02	98.47	429.1	1.50	3.09	0.34	1.60
	Bw	20–66	0.24	0.43	−0.94	4.24	1.38	41.0	1.00	9.61	97.58	437.0	1.50	9.21	0.17	1.20
	C	66–95	0.38	0.76	−1.29	0.22	0.63	32.0	23.0	12.22	95.63	450.1	1.60	14.3	0.17	1.40
HKuP05	A	0–23	0.32	0.71	−1.45	3.69	1.98	30.0	20.0	17.39	99.11	287.5	0.89	16.7	9.66	19.4
	Bw1	23–45	−0.13	0.13	−2.19	8.23	1.74	36.0	77.0	23.37	99.19	483.5	7.79	49.2	4.14	0.60
	Bw2	45–60	−0.01	0.17	−1.81	7.98	1.38	31.0	28.0	13.59	98.99	434.1	6.01	15.2	4.83	6.50
	C	60+	0.02	0.22	−1.93	7.98	1.27	39.0	31.0	14.13	99.00	495.4	5.54	24.2	6.21	3.10
HKuP06	A	0–5	0.60	0.47	−1.17	2.79	1.27	28.0	152.0	6.52	99.51	2760.7	0.33	9.43	9.66	20.0
	Bw1	5–20	0.75	1.07	−1.25	2.62	1.51	38.0	27.0	16.85	98.57	385.8	0.19	36.3	5.17	9.80
	Bw2	20–50	0.73	1.12	−1.37	2.45	1.51	35.0	27.0	21.74	98.07	285.2	0.18	48.5	4.83	39.2
	C1	50–62	0.73	1.18	−1.48	2.97	1.38	25.0	36.0	8.64	98.49	706.0	0.20	87.9	5.52	41.3
	C2	62+	0.63	1.15	−1.56	3.52	1.27	21.0	46.0	26.63	98.76	251.6	0.22	100.3	4.83	40.4
HKhP07	A	0–22	0.30	0.46	−1.28	1.92	1.51	25.0	23.0	12.22	97.52	392.8	16.5	2.50	0.86	10.7
	Bw	22–47	0.95	1.14	−0.23	2.45	0.19	35.0	16.0	12.69	96.35	401.9	14.8	2.40	0.86	12.8
	C	47–75	0.17	0.55	−1.50	2.97	1.38	27.0	54.0	12.22	97.66	662.9	14.8	1.90	5.17	13.8
	C2	75–110	0.24	0.70	−1.68	2.62	1.27	29.0	72.0	13.70	96.48	737.2	17.0	2.78	1.72	8.80
HKhP08	C	0–25	0.35	0.61	−1.35	3.34	1.51	40.0	3.00	11.33	95.35	379.5	30.0	3.40	0.17	12.0
	ABz	25–45	0.21	0.49	−1.34	4.24	1.38	41.0	1.00	14.15	97.85	296.8	70.5	4.54	0.34	31.6
	Cz	45–70	0.38	0.54	−1.20	0.22	0.63	32.0	23.0	14.65	98.52	375.4	82.0	6.32	0.52	46.2
	2Bwz	70–95	−0.22	−0.03	−1.40	0.23	0.65	24.0	34.0	14.15	97.43	409.9	78.1	11.2	0.69	35.5
	2Cz	95–120	−0.47	−0.09	−2.14	0.18	0.46	45.0	7.00	15.17	96.52	342.8	66.7	7.64	0.69	30.6
	2C2	120+	−1.10	−0.64	−3.02	0.15	0.42	38.0	5.00	15.17	97.55	283.5	71.4	6.90	0.17	33.3
HThP09	A	0–15	0.73	1.22	−1.41	0.27	1.92	23.0	117.0	31.52	97.00	444.2	0.26	55.7	9.31	25.2
	Bw1	15–35	0.73	1.30	−1.30	0.35	2.15	29.0	33.0	34.24	98.47	181.1	0.24	48.6	5.17	55.4
	Bw2	35–60	0.78	1.46	−1.15	0.82	1.21	29.0	117.0	33.70	99.53	433.2	0.23	48.9	4.48	100.0
	C1	60–85	0.84	1.57	−0.98	1.16	0.50	31.0	75.0	32.61	98.90	325.1	0.20	26.7	3.45	127.5
	C2	85+	0.95	1.67	−0.92	1.17	0.49	24.0	68.0	35.33	98.74	260.4	0.21	3.32	2.76	0.50
HThP10	A	0–15	−0.17	0.06	−2.44	43.2	2.34	59.0	39.0	14.13	99.45	693.6	15.3	27.2	15.5	32.0
	Bw1	15–35	0.00	0.24	−2.32	4.32	0.57	31.0	98.0	10.87	99.33	1186.8	8.36	26.0	13.1	21.8
	Bw2	35–50	−0.01	0.22	−2.27	2.36	0.73	21.0	53.0	12.50	98.72	592.0	8.76	26.6	10.7	41.7
HHuP11	A	0–10	−0.16	0.37	−1.94	38.9	1.27	159.0	23.0	27.72	99.64	656.6	16.3	46.2	5.25	30.7
	Bw1	10–38	0.00	0.63	−1.76	14.7	0.78	34.0	45.0	34.24	98.23	230.7	5.44	83.9	5.32	41.4
	Bw2	38–70	−0.70	0.15	−2.72	3.34	1.51	40.0	3.00	10.43	98.97	412.3	129.0	4.41	2.00	19.2
	C	70+	−0.85	0.03	−2.71	4.24	1.38	41.0	1.00	11.33	95.18	370.7	115.0	5.15	1.14	17.6
HHuP12	A	0–20	−0.47	0.12	−2.45	0.22	0.63	32.0	23.0	10.87	99.11	506.0	95.5	3.82	0.48	16.0
	Bw1	20–45	−0.45	0.06	−2.19	0.23	0.65	24.0	34.0	10.87	99.16	533.6	108.0	4.41	1.14	17.5
	Bw2	45–75	−0.31	−0.01	−2.33	0.18	0.46	45.0	7.00	11.33	97.59	459.0	100.0	7.64	2.34	15.0
	C	75+	0.54	0.74	−1.20	71.9	1.38	226.0	24.0	6.00	99.75	4166.7	1.10	1.18	1.38	1.40

BS = base saturation

a Cation exchange capacity by ammonium acetate at pH 7.

b By sum of cations.

c As (Ca + Mg/CEC)*100.

d Electrical conductivity.

e Calcium carbonate equivalent.

f Total organic matter calculated using the equation TOM = 1.724*TOC.

**Table 5:** Selective dissolutions analysis and P-retention in the selected representative profiles from different harrats of the Arabian Shield.

Profile	Horizon	Depth *Cm*	Al_p_	Fe_p_	Si_p_	Al_d_	Fe_d_	Al_o_	Fe_o_	Si_o_	P-retention
			g kg⁻^1^								%
HKP01	A	0–10	0.02	BDL	0.03	2.76	48.5	2.09	25.96	4.41	44.82
	Bw	10–25	0.04	0.01	0.07	2.99	49.76	2.42	23.23	4.37	44.62
	Bw1	25–55	0.03	BDL	0.05	2.97	49.19	2.71	22.56	4.51	45.22
	Bw2	55–80	0.02	BDL	0.03	3.80	52.78	3.46	11.34	4.26	45.32
HKP02	A	0–15	0.05	0.01	0.10	2.55	48.68	3.13	33.06	5.38	41.02
	Bw1	15–45	0.02	0.00	0.07	1.60	40.76	2.99	5.6	8.45	44.12
	Bw2	45–70	0.03	0.02	0.15	1.18	40.92	2.88	4.79	9.96	44.02
	C	70+	0.02	BDL	0.05	1.28	50.65	3.11	7.51	10.42	43.22
HRP03	A	0–10	0.04	0.01	0.09	1.33	39.93	2.91	5.27	8.23	43.90
	AB	10–40	0.05	0.05	0.17	0.91	40.09	2.80	4.46	9.74	43.80
	Bw	40–65	0.04	BDL	0.07	1.01	49.82	3.03	7.18	10.20	43.00
	Bw2	65–115	0.09	0.12	0.52	1.01	45.37	1.98	4.20	9.00	44.50
	C	115–130	0.05	BDL	0.06	2.45	47.63	1.95	25.59	4.13	44.52
HRP04	A	0–20	0.07	0.01	0.10	2.68	48.89	2.28	22.86	4.09	44.32
	Bw	20–66	0.06	BDL	0.08	2.66	48.32	2.57	22.19	4.23	44.92
	C	66–95	0.05	BDL	0.06	3.49	51.91	3.32	10.97	3.98	45.02
HKuP05	A	0–23	0.03	BDL	0.05	5.04	51.94	3.56	13.64	3.46	42.70
	Bw1	23–45	0.01	BDL	0.02	4.88	53.56	4.50	5.41	3.35	39.30
	Bw2	45–60	0.04	0.01	0.09	4.76	45.49	3.04	4.97	2.35	43.00
	C	60+	0.14	0.07	0.37	3.92	52.19	3.40	5.38	2.83	42.60
HKuP06	A	0–5	0.01	BDL	0.01	3.77	50.35	2.25	18.37	2.80	41.40
	Bw1	5–20	0.03	BDL	0.06	3.52	45.64	2.30	7.02	2.62	42.40
	Bw2	20–50	0.01	BDL	0.02	3.38	41.84	2.40	2.32	2.82	42.90
	C1	50–62	0.01	BDL	0.02	2.63	28.59	1.75	1.11	1.73	39.00
	C2	62+	0.01	BDL	0.02	2.52	26.93	1.15	0.25	0.85	43.40
HKhP07	A	0–22	0.03	BDL	0.04	2.51	47.70	1.99	25.76	4.29	44.67
	Bw	22–47	0.05	0.02	0.08	2.74	48.96	2.32	23.03	4.25	44.47
	C	47–75	0.04	BDL	0.06	2.72	48.39	2.61	22.36	4.39	45.07
	C2	75–110	0.03	BDL	0.04	3.55	51.98	3.36	11.14	4.14	45.17
HKhP08	C	0–25	0.06	0.03	0.11	2.30	47.88	3.03	32.86	5.26	40.87
	ABz	25–45	0.03	0.01	0.08	1.35	39.96	2.89	5.40	8.33	43.97
	Cz	45–70	0.04	0.03	0.16	0.93	40.12	2.78	4.59	9.84	43.87
	2Bwz	70–95	0.03	BDL	0.06	1.03	49.85	3.01	7.31	10.30	43.07
	2Cz	95–120	0.08	0.11	0.51	1.03	45.40	1.96	4.33	9.10	44.57
	2C2	120+	0.04	BDL	0.05	2.49	47.67	2.01	25.63	4.19	44.60
HThP09	A	0–15	0.03	BDL	0.06	4.91	48.40	4.62	8.70	5.65	42.90
	Bw1	15–35	0.02	BDL	0.05	5.00	55.27	4.62	11.99	5.51	45.60
	Bw2	35–60	0.01	BDL	0.01	5.64	60.08	4.31	11.52	5.26	44.30
	C1	60–85	0.01	BDL	0.01	5.97	59.62	4.33	8.88	4.42	44.50
	C2	85+	0.01	BDL	0.01	6.07	50.44	4.16	9.74	4.72	41.80
HThP10	A	0–15	0.00	BDL	0.01	3.48	39.35	3.80	22.66	6.57	45.30
	Bw1	15–35	0.01	BDL	0.01	3.27	40.09	3.90	27.76	7.40	46.10
	Bw2	35–50	0.02	BDL	0.02	2.89	37.02	3.07	25.55	6.64	43.00
HHuP11	A	0–10	0.03	BDL	0.06	1.26	50.62	3.13	7.38	10.32	43.15
	Bw1	10–38	0.08	0.10	0.51	1.26	46.17	2.08	4.40	9.12	44.65
	Bw2	38–70	0.04	BDL	0.05	2.70	48.43	2.05	25.79	4.25	44.67
	C	70+	0.06	0.01	0.09	2.93	49.69	2.38	23.06	4.21	44.47
HHuP12	A	0–20	0.05	BDL	0.07	2.91	49.12	2.67	22.39	4.35	45.07
	Bw1	20–45	0.04	BDL	0.05	3.74	52.71	3.42	11.17	4.10	45.17
	Bw2	45–75	0.07	0.01	0.12	2.49	48.61	3.09	32.89	5.22	40.87
	C	75+	0.04	0.00	0.09	1.54	40.69	2.95	5.43	8.29	43.97

Symbols: p = pyrophosphate extractable; o = oxalate extractable; d = dithionite extractable; BDL = below detectable limit.

**Table 6:** Selective dissolution ratios and allophane and ferrihydrite contents for selected representative profiles from different harrats of the Arabian Shield.

Profile	Horizon	Depth cm	(Al_o_-Al_p_)/Si_o_	Al_p_/Al_o_	Al_o_/Al_d_	Al_o_+1/2 Fe_o_	(Fe_d_ – Fe_o_) 100/Fe_d_	Fe_o_/Fe_d_	AP^a^	AP^b^	AP^c^	FH
			Ratio			%	Ratio		%			
HKP01	A	0–10	0.47	0.010	0.76	1.51	46.47	0.54	5.13	3.15	2.21	4.41
	Bw	10–25	0.54	0.017	0.81	1.40	53.32	0.47	4.38	3.12	2.19	3.95
	Bw1	25–55	0.59	0.011	0.91	1.40	54.14	0.46	4.15	3.22	2.26	3.84
	Bw2	55–80	0.81	0.006	0.91	0.91	78.51	0.21	2.88	3.04	2.13	1.93
HKP02	A	0–15	0.57	0.016	1.23	1.97	32.09	0.68	5.14	3.84	2.69	5.62
	Bw1	15–45	0.35	0.007	1.87	0.58	86.26	0.14	13.14	6.03	4.23	0.95
	Bw2	45–70	0.29	0.010	2.44	0.53	88.29	0.12	19.02	7.11	4.98	0.81
	C	70+	0.30	0.006	2.43	0.69	85.17	0.15	19.20	7.44	5.21	1.28
HRP03	A	0–10	0.35	0.014	2.19	0.55	86.80	0.13	12.90	5.88	4.12	0.90
	AB	10–40	0.28	0.018	3.08	0.50	88.88	0.11	18.85	6.95	4.87	0.76
	Bw	40–65	0.29	0.013	3.00	0.66	85.59	0.14	19.01	7.28	5.10	1.22
	Bw2	65–115	0.21	0.045	1.96	0.41	90.74	0.09	23.42	6.43	4.50	0.71
	C	115–130	0.46	0.026	0.80	1.47	46.27	0.54	4.91	2.95	2.07	4.35
HRP04	A	0–20	0.54	0.031	0.85	1.37	53.24	0.47	4.14	2.92	2.05	3.89
	Bw	20–66	0.59	0.023	0.97	1.37	54.08	0.46	3.90	3.02	2.12	3.77
	C	66–95	0.82	0.015	0.95	0.88	78.87	0.21	2.65	2.84	1.99	1.86
HKuP05	A	0–23	1.02	0.008	0.71	1.04	73.74	0.26	1.85	2.47	1.73	2.32
	Bw1	23–45	1.34	0.002	0.92	0.72	89.90	0.10	1.37	2.39	1.68	0.92
	Bw2	45–60	1.28	0.013	0.64	0.55	89.07	0.11	1.01	1.68	1.18	0.84
	C	60+	1.15	0.041	0.87	0.61	89.69	0.10	1.34	2.02	1.42	0.91
HKuP06	A	0–5	0.80	0.004	0.60	1.14	63.52	0.36	1.91	2.00	1.40	3.12
	Bw1	5–20	0.87	0.013	0.65	0.58	84.62	0.15	1.65	1.87	1.31	1.19
	Bw2	20–50	0.85	0.004	0.71	0.36	94.46	0.06	1.82	2.01	1.41	0.39
	C1	50–62	1.01	0.006	0.67	0.23	96.12	0.04	0.94	1.24	0.87	0.19
	C2	62+	1.34	0.009	0.46	0.13	99.07	0.01	0.35	0.61	0.43	0.04
HKhP07	A	0–22	0.46	0.015	0.79	1.49	46.00	0.54	5.13	3.06	2.15	4.38
	Bw	22–47	0.53	0.022	0.85	1.38	52.96	0.47	4.35	3.03	2.13	3.92
	C	47–75	0.59	0.015	0.96	1.38	53.79	0.46	4.10	3.13	2.20	3.80
	C2	75–110	0.80	0.009	0.95	0.89	78.57	0.21	2.81	2.96	2.07	1.89
HKhP08	C	0–25	0.56	0.020	1.32	1.95	31.37	0.69	5.09	3.76	2.63	5.59
	ABz	25–45	0.34	0.010	2.14	0.56	86.49	0.14	13.26	5.95	4.17	0.92
	Cz	45–70	0.28	0.014	2.99	0.51	88.56	0.11	19.31	7.03	4.92	0.78
	2Bwz	70–95	0.29	0.010	2.92	0.67	85.34	0.15	19.45	7.35	5.15	1.24
	2Cz	95–120	0.21	0.041	1.90	0.41	90.46	0.10	24.07	6.50	4.55	0.74
	2C2	120+	0.47	0.020	0.81	1.48	46.23	0.54	4.87	2.99	2.10	4.36
HThP09	A	0–15	0.81	0.006	0.94	0.90	82.02	0.18	3.80	4.03	2.83	1.48
	Bw1	15–35	0.83	0.004	0.92	1.06	78.31	0.22	3.61	3.93	2.76	2.04
	Bw2	35–60	0.82	0.002	0.76	1.01	80.83	0.19	3.52	3.76	2.63	1.96
	C1	60–85	0.98	0.002	0.73	0.88	85.11	0.15	2.47	3.16	2.21	1.51
	C2	85+	0.88	0.002	0.69	0.90	80.69	0.19	2.93	3.37	2.36	1.66
HThP10	A	0–15	0.58	0.000	1.09	1.51	42.41	0.58	6.21	4.69	3.29	3.85
	Bw1	15–35	0.53	0.003	1.19	1.78	30.76	0.69	7.69	5.28	3.70	4.72
	Bw2	35–50	0.46	0.007	1.06	1.58	30.98	0.69	7.90	4.74	3.32	4.34
HHuP11	A	0–10	0.30	0.010	2.48	0.68	85.42	0.15	18.77	7.37	5.16	1.25
	Bw1	10–38	0.22	0.038	1.65	0.43	90.47	0.10	22.73	6.51	4.56	0.75
	Bw2	38–70	0.47	0.020	0.76	1.49	46.75	0.53	4.91	3.03	2.13	4.38
	C	70+	0.55	0.025	0.81	1.39	53.59	0.46	4.17	3.01	2.11	3.92
HHuP12	A	0–20	0.60	0.019	0.92	1.39	54.42	0.46	3.95	3.11	2.18	3.81
	Bw1	20–45	0.82	0.012	0.91	0.90	78.81	0.21	2.72	2.93	2.05	1.90
	Bw2	45–75	0.58	0.023	1.24	1.95	32.34	0.68	4.93	3.73	2.61	5.59
	C	75+	0.35	0.014	1.92	0.57	86.66	0.13	12.91	5.92	4.15	0.92

Symbols: AP = Allophane content.

FH = Ferrihydrite content according to Parfitt and Childs (1988).

a according to Parfitt and Wilson (1985)

b according to Parfitt and Henmi (1982)

c according to Parfitt (1990).

**Table 7:** Analyzing the potential andic soil properties for selected representative profiles from different harrats of the Arabian Shield.

Profile	Horizon	Depth (cm)	FAF^a^ <2 mm	VG^b^	TOC^c^	Al_o_+1/2 Fe_o_	PR^d^	[(% Al_o_+1/2Fe_o_)*15.625)] +(%VG)	Soil classification
			%						Soil Taxonomy^e^
HKP01	A	0–10	83.24	15.00	0.42	1.51	44.82	38.59	Vitritorrands
	Bw	10–25	72.93	17.00	0.36	1.40	44.62	38.88	
	Bw1	25–55	54.97	17.00	0.32	1.40	45.22	38.88	
	Bw2	55–80	66.36	22.00	0.32	0.91	45.32	36.22	
HKP02	A	0–15	74.08	10.00	0.51	1.97	41.02	40.78	Haplotorrands
	Bw1	15–45	46.80	26.00	0.33	0.58	44.12	35.06	
	Bw2	45–70	66.68	29.00	0.20	0.53	44.02	37.28	
	C	70+	68.96	26.00	0.32	0.69	43.22	36.78	
HRP03	A	0–10	80.00	28.00	0.12	0.55	43.90	36.59	Vitritorrands
	AB	10–40	78.75	30.00	0.24	0.50	43.80	37.81	
	Bw	40–65	85.91	26.00	0.02	0.66	43.00	36.31	
	Bw2	65–115	77.50	30.00	0.01	0.41	44.50	36.41	
	C	115–130	88.75	15.00	0.02	1.47	44.52	37.97	
HRP04	A	0–20	50.00	15.00	0.02	1.37	44.32	36.41	Haplotorrands
	Bw	20–66	33.75	16.00	0.01	1.37	44.92	37.41	
	C	66–95	29.00	24.00	0.01	0.88	45.02	37.75	
HKuP05	A	0–23	43.31	20.00	0.56	1.04	42.70	36.30	Haplotorrands
	Bw1	23–45	70.07	27.00	0.24	0.72	39.30	38.30	
	Bw2	45–60	75.68	30.00	0.28	0.55	43.00	38.60	
	C	60+	73.77	28.00	0.36	0.61	42.60	37.50	
HKuP06	A	0–5	80.91	20.00	0.56	1.14	41.40	37.80	Haplotorrands
	Bw1	5–20	66.31	28.00	0.30	0.58	42.40	37.06	
	Bw2	20–50	63.28	35.00	0.28	0.36	42.90	40.60	
	C1	50–62	54.31	40.00	0.32	0.23	39.00	43.60	
	C2	62+	35.28	35.00	0.28	0.13	43.40	37.00	
HKhP07	A	0–22	35.00	12.00	0.05	1.49	44.67	35.28	Haplotorrands
	Bw	22–47	45.00	15.00	0.05	1.38	44.47	36.56	
	C	47–75	46.00	18.00	0.30	1.38	45.07	39.56	
	C2	75–110	45.50	24.00	0.10	0.89	45.17	37.91	
HKhP08	C	0–25	35.00	10.00	0.01	1.95	40.87	40.47	Haplotorrands
	ABz	25–45	30.00	28.00	0.02	0.56	43.97	36.75	
	Cz	45–70	15.00	29.00	0.03	0.51	43.87	36.97	
	2Bwz	70–95	35.00	27.00	0.04	0.67	43.07	37.47	
	2Cz	95–120	35.26	30.00	0.04	0.41	44.57	36.41	
	2C2	120+	61.50	13.00	0.01	1.48	44.60	36.13	
HThP09	A	0–15	52.58	25.00	0.54	0.90	42.90	39.10	Haplotorrands
	Bw1	15–35	59.08	22.00	0.30	1.06	45.60	38.60	
	Bw2	35–60	53.55	22.00	0.26	1.01	44.30	37.80	
	C1	60–85	53.51	25.00	0.20	0.88	44.50	38.80	
	C2	85+	50.05	23.00	0.16	0.90	41.80	37.10	
HThP10	A	0–15	52.38	20.00	0.90	1.51	45.30	43.60	Vitritorrands
	Bw1	15–35	54.27	15.00	0.76	1.78	46.10	42.80	
	Bw2	35–50	58.70	20.00	0.62	1.59	43.00	44.80	
HHuP11	A	0–10	68.00	26.00	0.30	0.68	43.15	36.63	Haplotorrands
	Bw1	10–38	58.60	30.00	0.31	0.43	44.65	36.72	
	Bw2	38–70	21.55	14.00	0.12	1.49	44.67	37.28	
	C	70+	34.00	16.00	0.07	1.39	44.47	37.72	
HHuP12	A	0–20	33.00	18.00	0.03	1.39	45.07	39.72	Haplotorrands
	Bw1	20–45	32.00	23.00	0.07	0.90	45.17	37.06	
	Bw2	45–75	35.00	7.00	0.14	1.95	40.87	37.47	
	C	75+	84.20	28.00	0.08	0.57	43.97	36.91	

a Fine earth fraction.

b Volcanic glass.

c Total organic carbon.

d Phosphorus retention; 100% of phosphate retention is equivalent to 5 g P kg^−1^ (air-dried soil).

e Soil Survey Staff (2014).

## 2 Experimental design, materials, and methods

The representative soil profiles were selected from six harrats within the Arabian Shield, Saudi Arabia. All profiles were excavated down to the C horizon and fully described in the field using the standard guidelines for soil profile description as outlined by Ref. [1]. Soil bulk density (BD) was determined using the core method [2]. Water retention capacity (at 33 and 1500 kPa) was determined by the pressure plate method [3]. Soils were size fractionated using the pipette method [4] and the percentage of sand, silt and clay fractions were used for soil texture identification using the USDA particle size classification [5]. Soil pH was measured potentiometrically using a pH meter (ORION STAR A211) in H_2_O, 1 M KCl, 0.01 M CaCl_2_, and 1 M NaF as outlined by Ref. [5]. The calcimeter method was used to determine the total CaCO_3_ equivalent [6]. Total organic carbon (TOC) was determined using the Walkley and Black wet digestion method [7]. Cation exchange capacity (CEC) and exchangeable cations were determined with extraction by the 1 M NH_4_OAc (pH = 7.0) method [8]. Base saturation was calculated from the sum of bases extracted by 1 M NH_4_OAc according to Ref. [8]. P-retention was determined using the Blakemore method [5] and measured using a spectrophotometer (Palintest 9100 UV-VIS, USA). Selective dissolution analyses were performed with acid ammonium oxalate (AAO), dithionite-citrate bicarbonate (DCB) and Na-pyrophosphate for the extractable Fe, Al, and Si [9], and measured using inductively coupled plasma optical emission spectroscopy (ICP-OES, Optima 4300 DV, PerkinElmer Inc). The allophane content was quantified using the methods of [10]. Ferrihydrite content was quantified according to Ref. [11]. Clay mineralogy was determined using an X-ray diffractometer (MAXima_X XRD-7000, Shimadzu, Japan) and interpreted according to Ref. [12]. The surface morphology of the clay minerals was investigated using scanning electron microscopy (SEM; EFI S50 Inspect, The Netherlands) and transmission electron microscopy (TEM 1011, Joel, Japan) according to Refs. [13,14]. Volcanic glass content was determined by the point count method using a petrographic microscope as described by Ref. [5]. The soils were classified based on their properties as described by Refs. [15,16].

## Transparency document

Transparency data associated with this article can be found in the online version.

## References

[1] Schoeneberger P.J., Wysocki D.A., Benham E.C., Broderson W.D. (2012). Field book for describing and sampling soils, Version 2.0. Natural Resources Conservation Service.

[2] Grossman R.B., Reinsch T.G., Dane H., Topp G.C. (2002). Bulk density and linear extensibility. Methods of Soil Analysis. Part 4. Physical Methods.

[3] Dane J.H., Hopmans J.W., Dane H., Topp G.C. (2002). Water retention and storage, laboratory methods. Methods of Soil Analysis. Part 4. Physical Methods.

[4] Gee G.W., Or D., Dane H., Topp G.C. (2002). Particle-size analysis. Methods of Soil Analysis. Part 4. Physical Methods.

[5] Burt R., Soil Survey Staff, Soil Survey Staff (2014). Kellogg soil survey laboratory methods manual. Soil Survey Investigations Report No. 42, Version 5.0.

[6] Loeppert R.H., Suarez D.L., Sparks D.L. (1996). Carbonate and gypsum. Methods of Soil Analysis. Part 3. Chemical Methods.

[7] Nelson D.W., Sommers L.E., Sparks D.L. (1996). Total carbon, organic carbon, and organic matter.

[8] Sumner M.E., Miller W.P., Sparks D.L. (1996). Cation exchange capacity and exchange coefficients. Methods of Soil Analysis. Part 3. Chemical Methods.

[9] Shang C., Zelazny L.W., Ulery A.L., Drees L.R. (2008). Selective dissolution techniques for mineral analysis of soils and sediments. Methods of Soil Analysis.

[10] Parfitt R.L., Wilson A.D. (1985). Estimation of allophane and halloysite in three sequences of volcanic soils, New Zealand. Catena.

[11] Parfitt R.L., Childs C.W. (1988). Estimation of forms of Fe and AI: a review, and analysis of contrasting soils by dissolution and Moessbauer methods. Aust. J. Soil Res..

[12] Moore D.M., Reynolds R.C. (1997). X-ray Diffraction and the Identification and Analysis of Clay Minerals.

[13] White G.N., Ulery A.L., Drees L.R. (2008). Scanning electron microscopy. Methods of Soil Analysis.

[14] Elsass F., Chenu C., Tessier, Ulery A.L., Drees L.R. (2008). Transmission electron microscopy for soil samples: preparation methods and use. Methods of Soil Analysis.

[15] Soil Survey Staff (2014). Keys to Soil Taxonomy.

[16] IUSS Working Group WRB (2015). International soil classification system for naming soils and creating legends for soil maps. WRB World Reference Base for Soil Resources. World Soil Resources Reports No. 106.

